# On the Modification Which the Blood Undergoes during Inflammation

**Published:** 1846-01

**Authors:** 


*On the Modification which the Blood undergoes during Inflammation.* By MM. Robert-Latour and Collignon.—The experiments of MM. Latour and Collignon were for the purpose of ascertaining the connection between the presence of fibrin in the blood and acute inflammation; and the investigations included the examination of arterial as well as of venous blood. These experimentalists drew, at the same moment, from a large dog, a quantity of arterial and of venous blood. The proportion of dry fibrin which the arterial blood furnished was found to be 0·20 per cent., while that of the venous blood was 0·25 per cent. Pleuro-pneumonic inflammation was then excited by the injection of a quantity of alcohol in the pleura; and, four days after, when the fever was high and the pulsation 190 in the minute, a second quantity of arterial and venous blood was again drawn. The arterial blood was now found to yield 0·40 per cent. of dry fibrin, and the venous 0·50 per cent.

A similar experiment was made on another dog with the same result, from which the authors conclude,

1st, That arterial, like venous blood, presents a notable increase of fibrin as soon as inflammatory action is set up.

2d, That the changes which the blood undergoes are the consequence, and not the cause of disease.—*From Comptes Rendus de Seances de l'Academie des Sciences, in Med. Examiner.*

